# CCL4 Deletion Accelerates Wound Healing by Improving Endothelial Cell Functions in Diabetes Mellitus

**DOI:** 10.3390/biomedicines10081963

**Published:** 2022-08-12

**Authors:** Ting-Ting Chang, Ching Chen, Liang-Yu Lin, Jaw-Wen Chen

**Affiliations:** 1 Department and Institute of Pharmacology, National Yang Ming Chiao Tung University, Taipei 11221, Taiwan; 2 School of Medicine, National Yang Ming Chiao Tung University, Taipei 11221, Taiwan; 3 Division of Endocrinology and Metabolism, Department of Medicine, Taipei Veterans General Hospital, Taipei 11217, Taiwan; 4 Healthcare and Services Center, Taipei Veterans General Hospital, Taipei 11217, Taiwan; 5 Division of Cardiology, Department of Medicine, Taipei Veterans General Hospital, Taipei 11217, Taiwan; 6 Cardiovascular Research Center, National Yang Ming Chiao Tung University, Taipei 11221, Taiwan

**Keywords:** chemokine CC motif ligand 4, diabetes mellitus, endothelial progenitor cells, human dermal microvascular endothelial cells, inflammation, wound healing

## Abstract

Chronic inflammation in diabetes mellitus (DM) is the leading cause of non-healing wounds. Chemokine CC motif ligand 4 (CCL4) is enhanced in the circulation and in the wounds of DM patients. This study aimed to investigate the effect of endogenous CCL4 inhibition on diabetic wound healing. Endothelial progenitor cells (EPCs) and human dermal microvascular endothelial cells (HDMECs) were used. Mice were injected with streptozotocin to generate hyperglycemia. An enhanced CCL4 level as well as decreased tube formation and migration abilities were observed in high-glucose-treated HDMECs and in EPCs from type 2 DM patients. CCL4 inhibition by siRNA restored the damaged cell function by upregulating the Akt/endothelial nitric oxide synthase/vascular endothelial growth factor/stromal cell-derived factor-1α pathways. Wild-type diabetic mice had delayed wound repair, whereas the CCL4-knockout diabetic mice showed an accelerated rate of wound closure. In a Matrigel plug assay, CCL4-knockout diabetic mice showed higher blood vessel and hemoglobin levels. Higher CD31 and Ki67 expression in the wound area and Matrigel plugs was detected in the CCL4-knockout diabetic mice. CCL4-knockout mice had upregulated angiogenic factors and downregulated inflammatory factors. This study might provide the theoretical basis for CCL4 inhibition as a therapeutic option for clinical diabetic wound treatment.

## 1. Introduction

Diabetes mellitus (DM) is a chronic metabolic syndrome, which is mainly characterized by insufficient insulin secretion or functional impairment, resulting in long-term hyperglycemia and chronic inflammation [1]. Chronic inflammation leads to diabetic vasculopathy and diabetic foot ulcers in both type 1 DM and type 2 DM [2]. Delayed wound healing in DM results from a complex pathophysiology involving blood vessels, neuropathy, and immunity. The pathological wound repair is mainly involved in impaired endothelial cell function [3]. Meanwhile, neutrophils and macrophages rapidly infiltrate the diabetic wound and release cytokines and chemokines to generate an inflammatory environment [4,5]. Then, macrophages and the inflammatory environment can promote matrix metalloproteinase (MMP) production, cause extracellular matrix imbalance, damage new granulation tissue, and impair vascular endothelial cell function [5,6]. Accordingly, management of the inflammation and amelioration of the vascular endothelial cell function might be the key to accelerating diabetic wound healing.

Chemokine CC motif ligand (CCL) 4 is a member of the CC chemokine family [7]. CCL4 plays a role in the chemotactic activity of immune cells [8,9,10]. CCL4 can induce the production of reactive oxygen species (ROS) in endothelial cells [11], and it is associated with atherosclerosis and cardiovascular disease [12,13]. The inhibition of CCL4 by neutralizing antibodies improved ischemia-induced angiogenesis in both type 1 and type 2 diabetic as well as metabolic syndrome mice [14]. Given that CCL4 expression may be increased in patients with type 1 and type 2 DM and in their wounds [5,15], it is interesting to further explore whether an anti-CCL4 strategy might have beneficial effects on diabetic wound healing. Accordingly, we sought to use both in vitro and in vivo experiments to prove that anti-CCL4 could enhance diabetic wound healing by improving endothelial cell function. The findings of this study clarify whether the deletion of CCL4 can accelerate diabetic wound healing by improving endothelial function.

## 2. Materials and Methods

### 2.1. Cell Culture

A blood sample was collected from the peripheral veins of diabetic patients and healthy volunteers. After the blood was collected, the total mononuclear cells were separated by Histopaque-1077 (Sigma-Aldrich, 10771, Darmstadt, Germany) and centrifuged at 500× *g* at room temperature for 30 min. The mononuclear cells were cultured in Endothelial Cell Growth Basal Medium-2 (Lonza, Catalog #00190860, Basel, Switzerland) with supplements and 20% fetal bovine serum on fibronectin-coated 6-well plates. After a 4-day culture, the medium was removed, leading to removed non-adherent cells and attached early endothelial progenitor cells (EPCs) in the shape of an elongated spindle. Then, after being cultured for 2–4 weeks, attached late EPCs emerged. Late EPCs were in the shape of a cobblestone, and this kind of shape is the typical monolayer growth pattern of mature endothelial cells. Late EPCs were cultured with Endothelial Cell Growth Basal Medium-2 containing 10% fetal bovine serum and 1% penicillin/streptomycin (Sigma-Aldrich, P4333, Darmstadt, Germany), Human dermal microvascular endothelial cells (HDMECs, ScienCell, Catalog #2000, Carlsbad, CA, USA) were cultured with Endothelium Cell Medium containing 5% fetal bovine serum, VEGF, and 1% penicillin/streptomycin (Sigma-Aldrich, P4333, Darmstadt, Germany), and the cultured dishes were coated with fibronectin before being used. The human study was approved by the institute’s research committee and conformed to the Declaration of Helsinki.

### 2.2. Transfection of CCL4 siRNA

Cells were transfected with CCL4 siRNA (Santa Cruz, sc-43932, Dallas, TX, USA) using Oligofectamine (Invitrogen, 12252011, Carlsbad, CA, USA) in opti-MEM. The final concentration of siRNA was 80 nM. After transfection, the cells were treated with a high concentration of glucose (25 mM) for 2 days. Then, cells were collected for the next experiments.

### 2.3. Migration Assay

The Transwell migration assay was used to analyze the migrating ability of late EPCs or HDMECs after treatments. The cells (2 × 10^4^ or 2 × 10^5^ cells) were suspended in a serum-free cultured medium. HDMECs were incubated with a medium containing 25 mM glucose for 2 days after treatment. The cells were seeded on the upper chamber of a 24-well Transwell plate with a polycarbonate membrane, and the cells migrated toward the lower chamber containing 400 μL cultured medium with 10% fetal bovine serum at 37 °C and 5% CO_2_. After 18 h, the migrated cells were fixed in 4% paraformaldehyde and stained with hematoxylin solution. Images were captured using a high-power (×100) microscope (Nikon, Eclipse TS100, Tokyo, Japan).

### 2.4. Tube Formation Assay

Late EPCs or HDMECs were seeded into a 6-well plate in each well until a monolayer was formed, and the combined treatment was then conducted. Cells were collected by trypsinization, and 2 × 10^4^ cells/well were seeded into ECMatrix gel (Invitrogen, Carlsbad, CA, USA) in 96-well plates, in 100 μL cultured medium with 10% FBS, for 16 h at 37 °C and 5% CO_2_. Images were captured using a high-power (×40) microscope (Nikon, Eclipse TS100, Tokyo, Japan). The numbers of formed tubes of cells were calculated using Image-Pro Plus computer software (Media Cybernetics, Inc., Rockville, MD, USA).

### 2.5. Western Blot

Total proteins were extracted using lysis buffer (25 mM Tris, 150 mM sodium chloride, 1% NP-40, 1% sodium deoxycholate, 0.1% SDS, pH 7.6), and the proteins were separated in 8–12% (*v*/*v*) SDS-PAGE gels. After electrophoresis (Bio-Rad Laboratories, Hercules, CA, USA), the proteins were transferred onto nitrocellulose membranes (Millipore, Darmstadt, Germany), and the membrane was incubated with anti-vascular endothelial growth factor (VEGF) antibody (Cell Signaling Technology, #2463, 1:1000, Danvers, MA, USA), anti-stromal cell-derived factor (SDF)-1α (Cell Signaling Technology, #3530, 1:1000, Danvers, MA, USA), anti-phospho-endothelial nitric oxide synthase (eNOS) (Cell Signaling, #9571, 1:1000, Danvers, MA, USA/Genetex, GTX129058,1:1000, Irvine, CA, USA), eNOS (Cell Signaling, #32027, 1:1000, Danvers, MA, USA/Genetex, GTX50505, 1:1000, Irvine, CA, USA), phospho-AKT (BD Biosciences, 550747, 1:1000, Franklin Lakes, NJ, USA), AKT (BD Biosciences, 610868, 1:1000, Franklin Lakes, NJ, USA), anti-CCL4 (Santa Cruz, sc-393441, 1:1000, Dallas, TX, USA), anti-tumor necrosis factor (TNF)-α (Cell Signaling Technology, #3707,1:1000, Danvers, MA, USA), IL-6 (Cell Signaling Technology, #1215,1:1000, Danvers, MA, USA), and anti-actin (Cell Signaling Technology, #3700, 1:10,000, Danvers, MA, USA) at 4 °C overnight. After washing three times, the membranes were incubated with HRP-conjugated secondary antibodies (1:1000) for 1 h at room temperature. Finally, the membranes were visualized using an ECL kit. Each group was corrected with a control, and the control value of each experiment was represented as one-fold in the Western blot quantitative analysis.

### 2.6. Animal Preparation

Six-week-old male C57BL/6JNarl-Ccl4em1 knockout (CCL4KO) mice were designed and purchased from the National Laboratory Animal Center (Taipei, Taiwan). CCL4KO mice were generated in a C57BL/6JNarl genetic background by using the CRISPR/Cas9 system. All mice were genotyped using PCR with specific primers (forward, 5′-TCTCCCTCCTTTCTCTTCCGTG-3′; reverse, 5′-TCTACTCCCAATGATGGCTGACC-3′). C57BL/6JNarl mice were used as the wild-type (WT) control. Mice were raised under specific pathogen-free conditions and were kept in microisolator cages with 12:12-h light/dark cycles and free access to water and standard mouse chow.

To generate hyperglycemia, some mice were intraperitoneally injected with streptozotocin (STZ) at 40 mg/kg for 5 days. Hyperglycemia was defined as blood sugar levels higher than 250 mg/dL. The following experiments were conducted after two weeks of stabilization after hyperglycemia. The timepoint to test vascular complications was used from a previous study [14]. The experiments described here were approved by the Institutional Animal Care and Use Committee (IACUC) of National Yang Ming Chiao Tung University (Taipei, Taiwan).

### 2.7. Wound Healing Assay

Mice were anesthetized with 1% isoflurane. The back skin was shaved and cleaned with 75% alcohol. Circular, full-thickness excisional wounds of 3 mm in diameter were generated by biopsy punch without muscle injury. The wounds were recorded using a digital camera (Nikon, Tokyo, Japan) at 0, 1, 3, 5, and 7 days after the wounds were generated.

### 2.8. Matrigel Plug Assay

The mice were injected subcutaneously with a growth factor-reduced basement membrane matrix (Corning^®^ Matrigel, 356231, Glendale, AZ, USA) containing 30 ng/mL VEGF and 50 U/mL heparin (Sigma-Aldrich, H3393, Darmstadt, Germany). The gel formed a solid plug as it touched the body temperature. After 14 days, the plugs were collected and homogenized using 500 μL cell lysis buffer and centrifuged at 6000× *g* at 4 °C for 1 h. A colorimetric assay (Sigma-Aldrich, MAK115, Darmstadt, Germany) was used to detect hemoglobin using a microplate reader at 400 nm wavelength. The plug was harvested for histological and immunohistochemistry analysis.

### 2.9. Mouse Blood Glucose Test

After fasting for 4 h, 1 μL of mouse blood was collected from the tail. An Abbott FreeStyle glucometer (Abbot, OPTIUM XCEED, Chicago, IL, USA) was used following the instructions provided by the original manufacturer.

### 2.10. Histological and Immunohistochemistry Analysis

The wound sample was fixed with 4% paraformaldehyde for 24 h; the sample was dehydrated in graded alcohols and then embedded in paraffin wax. The tissues were sectioned into samples of 5 μm thickness. The sections were dried overnight and stained with hematoxylin and eosin (H&E) for histological analysis. The paraffin wax-embedded tissues were sectioned to 5 μm thickness and rehydrated. Antigen retrieval was performed by using 0.05 M sodium citrate buffer. The slides were then incubated at 4 °C overnight with a primary antibody to detect CD31 (Abcam, 28364, 1:100 Waltham, MA, USA) and Ki67 (Novus, NB500-170, 1:100 Littleton, CO, USA). The sample was washed with PBS solution and incubated with a secondary antibody (rabbit; 1:1000) for 2 h at room temperature.

### 2.11. Evaluation of VEGF and SDF-1α Concentrations

The serum concentrations of VEGF and SDF-1α were determined by ELISA (R&D, MMV00 and MCX120, Minneapolis, MN, USA) according to the manufacturer’s instructions.

### 2.12. Statistics

The results are presented as the mean ± standard deviation. Statistical analysis was performed using an unpaired Student’s *t*-test for analysis of variance and MANOVA for validation. Then, Scheffe’s method, a post hoc test for multiple comparisons, was used. SPSS software (version 14; SPSS, Chicago, IL, USA) was used to analyze the data. A *p*-value < 0.05 was considered statistically significant.

## 3. Results

### 3.1. Knockdown of CCL4 Ameliorated Cell Function in HG-Stimulated HDMECs

**Figure 1:**
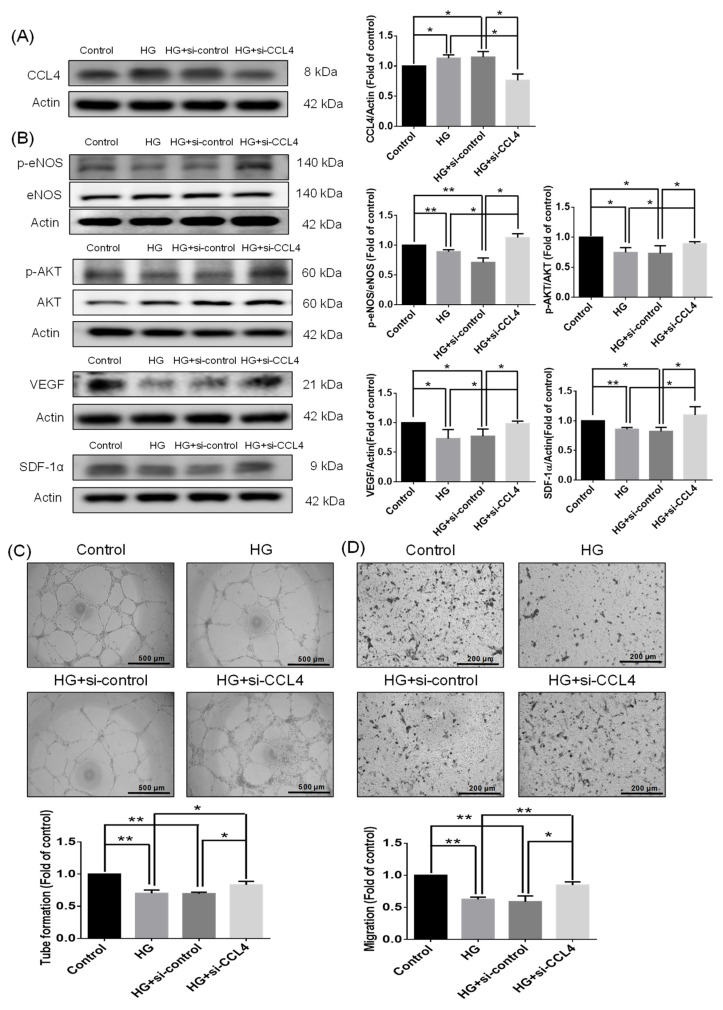
CCL4 inhibition by siRNA recovered functions of high-glucose-impaired HDMECs with increased angiogenic protein expressions. Western blotting and statistical analyses of CCL4 ((**A**); *n* = 3), p-AKT, p-eNOS, VEGF, and SDF-1α. The angiogenic proteins were reversed after the administration of siCCL4 ((**B**); *n* = 3). Tube formation ability and quantitative analysis of HDMECs; the images were captured using a (×40) microscope. The tube formation ability was improved after the administration of siCCL4 ((**C**); *n* = 3). Migration ability and quantitative analysis of HDMECs; the images were captured using a (×100) microscope. The migration ability was improved after the administration of siCCL4 ((**D**); *n* = 3). HG represents high glucose. * *p* < 0.05, ** *p* < 0.01.

HDMECs isolated from adult skins were used to explore the direct effects of CCL4 in the wound healing process under the pathological condition in vitro. CCL4 was upregulated in the HG-stimulated HDMECs and was knocked down by administration of CCL4 siRNA (Figure 1A). The protein expressions of p-Akt, p-eNOS, VEGF, and SDF-1α were downregulated in the HG-stimulated HDMECs and were reversed in the CCL4 knockdown group (Figure 1B). The HDMECs showed impaired tube formation and migration abilities under the HG conditions, and these abilities were improved by the administration of CCL4 siRNA (Figure 1C,D). These results suggest that the knockdown of CCL4 could ameliorate HDMEC function by upregulating angiogenic protein expressions such as AKT/eNOS/VEGF/SDF-1α under the HG condition.

### 3.2. Knockdown of CCL4 Improved the Cell Function of EPCs from Patients with Type 2 DM

**Figure 2:**
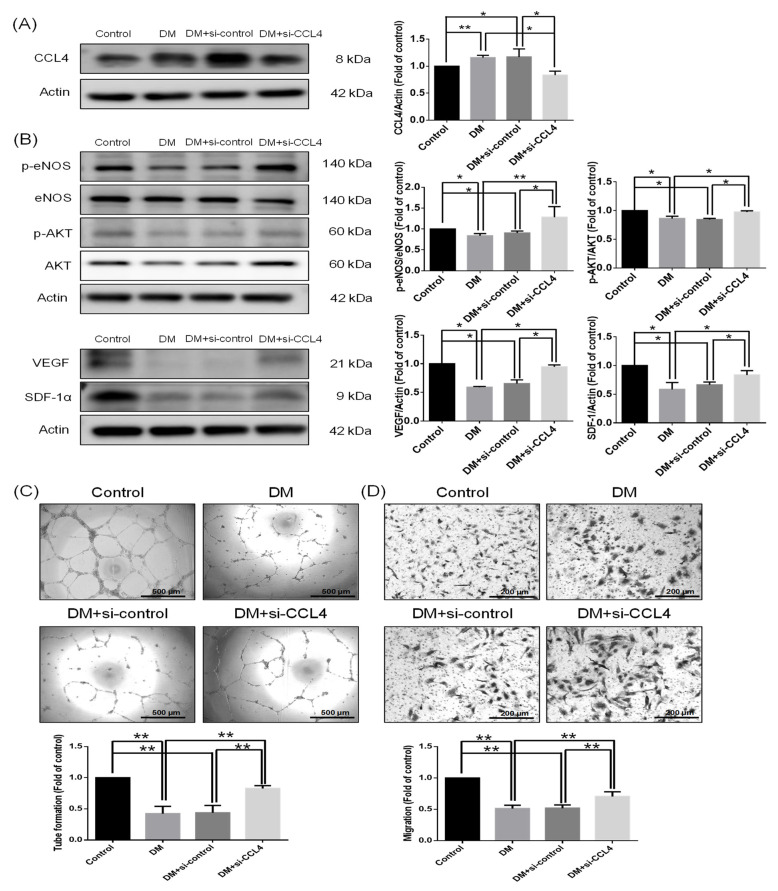
CCL4 inhibition by siRNA recovered functions of EPCs from type 2 DM patients with increased angiogenic protein expressions. Western blotting and statistical analyses of CCL4 ((**A**); *n* = 3), p-AKT, p-eNOS, VEGF, and SDF-1α. The angiogenic proteins were reversed after the administration of siCCL4 ((**B**); *n* = 3). Tube formation ability and quantitative analysis of EPCs from type 2 DM patients and control subjects; the images were captured using a (×40) microscope. The tube formation ability was improved after the administration of siCCL4 ((**C**); *n* = 3). Migration ability and quantitative analysis of EPCs from type 2 DM patients and control subjects; the images were captured using a (×100) microscope. The migration ability was improved after the administration of siCCL4 ((**D**); *n* = 3). * *p* < 0.05, ** *p* < 0.01.

CCL4 expression was higher in the EPCs from type 2 DM patients compared to those from the control volunteers. The enhanced CCL4 expression was knocked down by the administration of CCL4 siRNA in the EPCs from type 2 DM patients (Figure 2A). The protein expressions of p-Akt, p-eNOS, VEGF, and SDF-1α were decreased in the EPCs from type 2 DM patients and were enhanced in the CCL4 knockdown group (Figure 2B). Both tube formation and migration abilities were impaired in the EPCs from type 2 DM patients, and these abilities were improved in the CCL4 knockdown group (Figure 2C,D). These results demonstrate that the knockdown of CCL4 could improve the functions of EPCs from type 2 DM patients by upregulating angiogenic protein expressions such as AKT/eNOS/VEGF/SDF-1α.

### 3.3. Deletion of CCL4 Accelerated Wound Repair in Diabetic Mice

**Figure 3:**
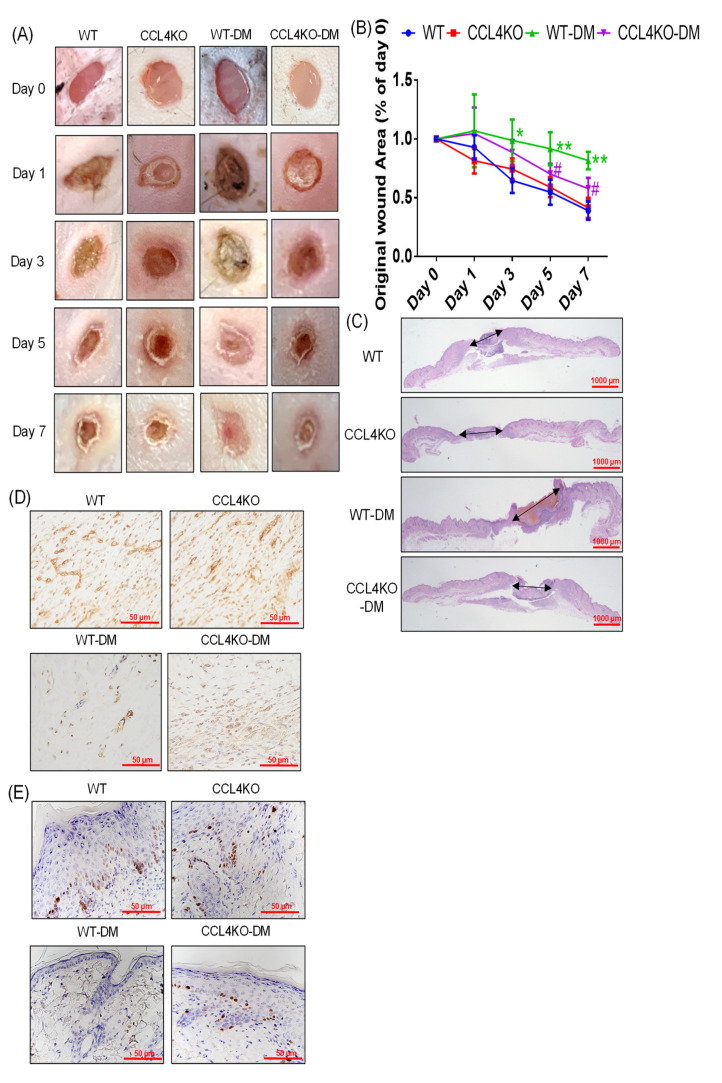

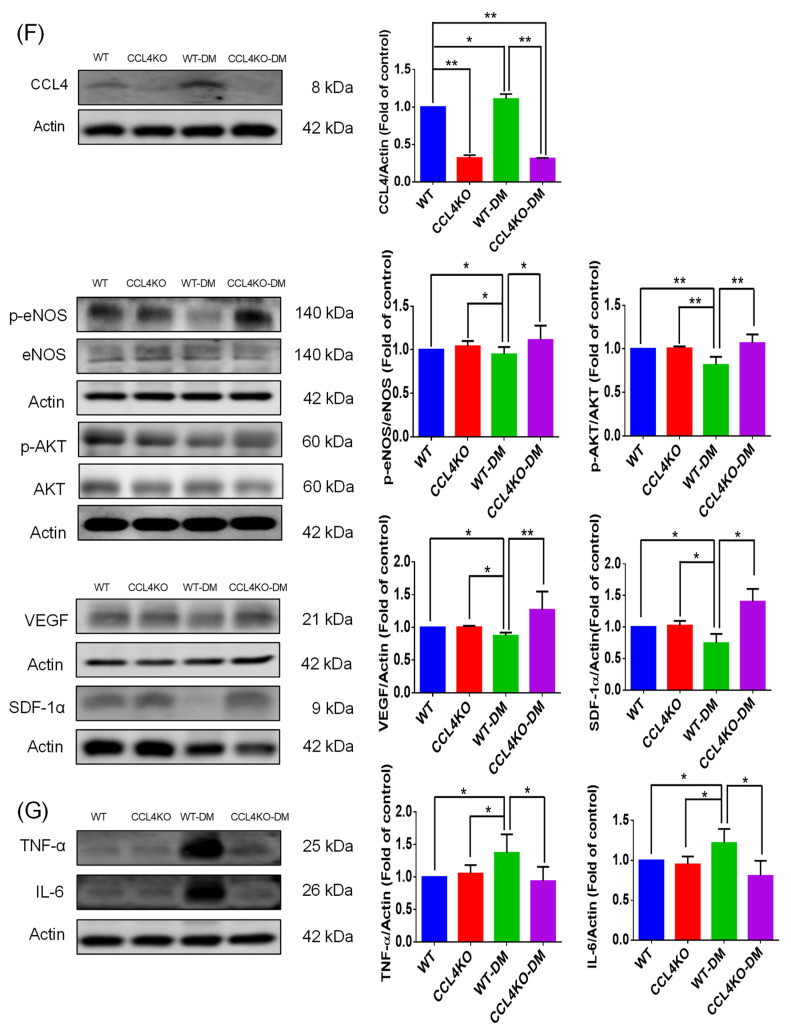
CCL4 inhibition by knockout improved wound repair in diabetic mice. Representative wound areas (**A**). The closure rates of 3-millimeter punch biopsies were measured ((**B**); *n* = 6). Representative images with H&E staining (**C**). Representative images with immunostaining of CD31 and Ki67. Both CD31- and Ki67-positive areas were enhanced in the CCL4-knockout mice (**D**,**E**). Western blotting and statistical analyses of CCL4, p-AKT, p-eNOS, VEGF, and SDF-1α in the wound area. The angiogenic proteins were enhanced in tissues from the CCL4-knockout mice ((**F**); *n* = 3). Western blotting and statistical analyses of TNF-α and IL-6 in the wound area. The inflammatory proteins were decreased in tissues from the CCL4-knockout mice ((**G**); *n* = 3). WT, wild-type mice; CCL4KO, CCL4-knockout mice; WT-DM, wild-type diabetic mice; CCL4KO-DM, CCL4-knockout diabetic mice. * *p* < 0.05, ** *p* < 0.01 compared with WT, # *p* < 0.05 compared with WT-DM.

The WT diabetic mice had significantly delayed wound repair on day 3 post-injury compared to the WT group. The CCL4KO diabetic mice showed an accelerated rate of wound closure on days 5 and 7 post-injury compared to the WT diabetic mice (Figure 3A,B). The improved wound healing in the CCL4KO diabetic mice was also observed in the wound sections analyzed by H&E staining (Figure 3C). Higher CD31 and Ki67 expressions in the wound area were detected in the CCL4KO diabetic mice than in the WT diabetic mice (Figure 3D,E). Moreover, the protein expressions of p-Akt, p-eNOS, VEGF, and SDF-1α were decreased in the wound area of the WT diabetic mice compared to that of the WT group. These proteins were all enhanced in the CCL4KO diabetic mice and not in the WT diabetic mice (Figure 3F). On the other hand, the protein expressions of TNF-α and IL-6 were enhanced in the wound area of the WT diabetic mice and were reduced in the CCL4KO diabetic mice (Figure 3G). These results suggest that CCL4 knockout could promote wound closure in diabetic mice by enhancing angiogenesis and decreasing inflammation.

### 3.4. Deletion of CCL4 Promoted Neovascularization in Diabetic Mice

**Figure 4:**
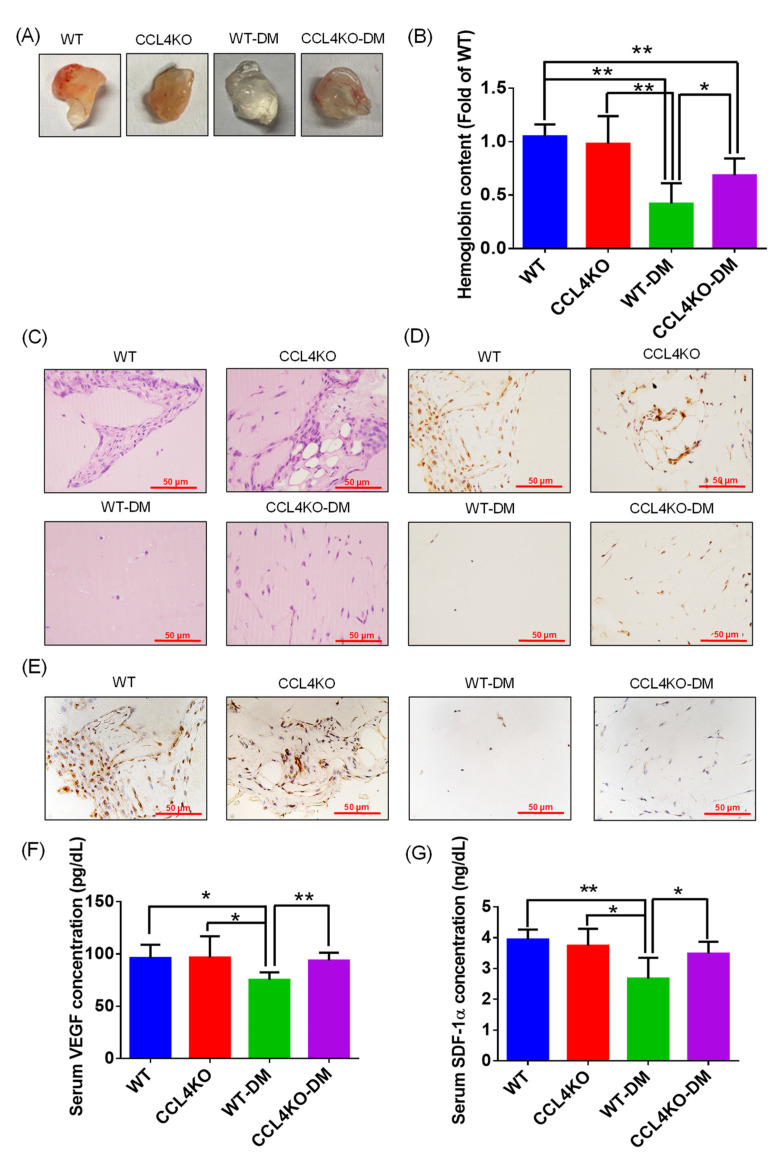
CCL4 inhibition by knockout enhanced neovascularization in diabetic mice. Representative Matrigel plug (**A**) and analysis of hemoglobin content ((**B**); *n* = 6). Representative images with H&E staining (**C**). Representative images with immunostaining of CD31 and Ki67. Both CD31- and Ki67-positive areas were enhanced in the CCL4-knockout mice (**D**,**E**). Serum concentrations of VEGF and SDF-1α were higher in the CCL4-knockout diabetic mice than in the wild-type diabetic mice ((**F**,**G**); *n* = 6). WT, wild-type mice; CCL4KO, CCL4-knockout mice; WT-DM, wild-type diabetic mice; CCL4KO-DM, CCL4-knockout diabetic mice. * *p* < 0.05, ** *p* < 0.01.

A Matrigel plug assay was performed to explore the effects of CCL4 knockout on new blood vessel forming ability in diabetic mice. The blood vessel and hemoglobin contents were reduced in the WT diabetic mice compared to the WT mice. The CCL4KO diabetic mice showed higher blood vessel and hemoglobin levels compared to the WT diabetic mice (Figure 4A,B). The increased ability to form new blood vessels in the CCL4KO diabetic mice was also observed in the Matrigel plug sections analyzed by H&E staining (Figure 4C). Higher CD31 and Ki67 expressions in the section were detected in the CCL4KO diabetic mice than in the WT diabetic mice (Figure 4D,E). The serum levels of VEGF and SDF-1α were reduced in the WT diabetic mice and were enhanced in the CCL4KO diabetic mice (Figure 4F,G). These results reveal that CCL4 knockout could enhance the neovascularization ability in diabetic mice.

## 4. Discussion

The study has several fundamental findings. First, impaired cell function and higher expression of CCL4 were observed in HG-stimulated HDMECs and EPCs from type 2 DM patients. CCL4 knockdown could reverse cell dysfunction with upregulated Akt/eNOS/VEGF/SDF-1α pathways. Second, delayed wound healing was observed in diabetic animals with enhanced CCL4 protein levels. CCL4-knockout diabetic animals showed accelerated wound repair with higher levels of capillary density and cell proliferation. Third, CCL4-knockout diabetic animals showed increased neovascularization with higher levels of hemoglobin, capillary density, and cell proliferation. Consistent with the in vitro findings, the improvement of wound repair as well as neovascularization was accompanied by upregulated Akt/eNOS/VEGF/SDF-1α protein expression in vivo. Furthermore, inflammatory factors including TNF-α and IL-6 were downregulated with the accelerated wound healing in CCL4-knockout diabetic animals.

Inflammatory factors can be significantly increased in the circulation and in the wound areas of patients with diabetes, causing a long-term inflammatory response [16]. Chronic inflammation attenuates the activation of the Akt/eNOS signaling pathways, increases inducible nitric oxide synthase expression, and decreases nitric oxide release, resulting in decreased EPC and endothelial cell numbers and damaged cell function [17,18,19,20]. Acarbose promoted wound healing and improved angiogenesis in diabetic mice through the Akt/eNOS signaling pathway [21]. Although an increase in CCL4 has been observed in the wounds of diabetic patients [5], there have been no experiments exploring the effects of anti-CCL4 as a potential treatment option. We previously showed that EPCs from type 2 DM patients could secrete more CCL4 than those from healthy subjects. Exogenous CCL4 inhibition by neutralizing antibodies could improve EPC function by sensitizing CXCR4 expression and increasing angiogenic proteins such as VEGF and SDF-1α [14]. In line with our previous findings, the present study further showed that endogenous CCL4 inhibition by siRNA could upregulate the Akt/eNOS/VEGF/SDF-1α pathways in both HG-stimulated HDMECs and EPCs from type 2 DM patients. Additionally, compared to wild-type mice, the CCL4-knockout mice showed rapid diabetic wound repair with enhanced angiogenesis. Taken together, one may speculate that endogenous CCL4 inhibition might facilitate the angiogenesis of the wound area by improving endothelial cell function.

The diabetic wounds usually had a higher expression of inflammatory cytokines/chemokines and a lower expression of anti-inflammatory cytokines [5,22]. Modulation of inflammation including cytokines and chemokines could affect the healing process of diabetic wounds. Hypoxia adipose stem cell-derived exosomes could promote the healing of a diabetic wound by inhibiting inflammation through the PI3K/AKT signaling pathway [23]. IL-6 expression increased in the skin of diabetes patients [24]. Inhibition of glycation product receptors reduced TNF-α and IL-6 and accelerated epithelialization and angiogenesis to promote diabetic wound closure [25]. Inhibition of SDF-1α resulted in a decrease in the rate of diabetic wound healing with increased pro-inflammatory gene expression of IL-6 [26]. On the other hand, negative pressure wound therapy promoted wound healing with decreased inflammatory factors such as TNF-α and IL-6 in clinical patients [27]. Furthermore, inhibition of TNF-α was shown to be one of the anti-inflammatory strategies that could enhance diabetic wound angiogenesis and healing with increased VEGF receptor 2 [28]. Anti-CCL17/22 also accelerated diabetic wound healing with fewer regulatory T cells in the wound bed [29]. In contrast, topical application of CCL2 and CCL3 could promote diabetic wound closure [30,31]. It seems that anti-inflammation could be an optimal strategy to accelerate wound healing. We previously showed that inhibition of CCL4 by antibodies could decrease the circulating and pancreatic expression of TNF-α and IL-6 in diabetic mice [32]. Here, we further demonstrated that the genetic knockout of CCL4 could also provide protective effects on diabetic wounds accompanied with downregulated inflammatory factors.

There are some issues that should be further addressed. Firstly, we have recently shown that the inhibition of CCL4 by the CCL4 antibody could protect pancreatic islets and stabilize glucose metabolism in diabetic mice [32]. In a diet-induced DM model, CCL4-knockout mice had improved blood sugar levels in the oral glucose tolerance tests as well as lower homeostasis in the model assessment of insulin resistance [33]. In the current study, while diabetes was well established and hyperglycemia was confirmed in the CCL4-knockout mice, there were no differences in the fasting blood glucose between the CCL4-knockout diabetic mice and the wild-type diabetic mice. We cannot exclude the potential benefits of glucose control in wound healing in vivo. However, the in vitro CCL4 knockdown could improve endothelial cell migration and tube formation, suggesting direct beneficial effects of CCL4 inhibition on angiogenesis for wound healing. Secondly, wound healing is a complex process. Mechanisms other than angiogenesis and inflammation, such as MMP expression, etc., may also contribute to wound healing. While both angiogenesis and inflammation were improved by either CCL4 knockdown in vitro, CCL4 knockout in vivo, or both, further studies may be required to see if CCL4 inhibition could modulate other mechanisms contributing to wound healing. In addition, the double staining of CD31 and Ki67 should be further explored in the future to identify the proliferating cells. Finally, while CCL4 is a chemokine involving universal inflammation [10], investigations on other more specific mechanisms may still be required to further promote wound healing.

In conclusion, higher CCL4 expression and impaired cell function were observed in HG-treated HDMECs and in EPCs from type 2 DM patients. Endogenous CCL4 inhibition could restore the damaged cell function by upregulating the Akt/eNOS/VEGF/SDF-1α pathways. In diabetic mice, deletion of CCL4 accelerated wound healing and neovascularization by upregulating angiogenic factors such as VEGF and SDF-1α as well as downregulating inflammatory factors such as TNF-α and IL-6. Therefore, the inhibition of CCL4 facilitated angiogenesis and attenuated the inflammatory response, promoting healing in the wound area in diabetic animals. This study might provide the theoretical basis for CCL4 inhibition as a potential therapeutic option for clinical diabetic wound treatment.
